# Heterogeneous glioblastoma cell cross-talk promotes phenotype alterations and enhanced drug resistance

**DOI:** 10.18632/oncotarget.5701

**Published:** 2015-10-20

**Authors:** Helena Motaln, Ana Koren, Kristina Gruden, Živa Ramšak, Christian Schichor, Tamara T. Lah

**Affiliations:** ^1^ Department of Genetic Toxicology and Cancer Biology, National Institute of Biology, Ljubljana, Slovenia; ^2^ Laboratory for Clinical Immunology and Molecular Genetics, University Clinic Golnik, Golnik, Slovenia; ^3^ Department of Biotechnology and Systems Biology, National Institute of Biology, Ljubljana, Slovenia; ^4^ Department of Neurosurgery, Klinikum GroΔhadern, Ludwig-Maximilians-Universität, Munich, Germany; ^5^ Department of Biochemistry, Faculty of Chemistry and Chemical Engineering, University of Ljubljana, Ljubljana, Slovenia

**Keywords:** glioblastoma heterogeneity, U87 cells, temozolomide resistance, cellular cross-talk, transcriptomics

## Abstract

Glioblastoma multiforme is the most lethal of brain cancer, and it comprises a heterogeneous mixture of functionally distinct cancer cells that affect tumor progression. We examined the U87, U251, and U373 malignant cell lines as *in vitro* models to determine the impact of cellular cross-talk on their phenotypic alterations in co-cultures. These cells were also studied at the transcriptome level, to define the mechanisms of their observed mutually affected genomic stability, proliferation, invasion and resistance to temozolomide. This is the first direct demonstration of the neural and mesenchymal molecular fingerprints of U87 and U373 cells, respectively. U87-cell conditioned medium lowered the genomic stability of U373 (U251) cells, without affecting cell proliferation. In contrast, upon exposure of U87 cells to U373 (U251) conditioned medium, U87 cells showed increased genomic stability, decreased proliferation rates and increased invasion, due to a plethora of produced cytokines identified in the co-culture media. This cross talk altered the expression 264 genes in U87 cells that are associated with proliferation, inflammation, migration, and adhesion, and 221 genes in U373 cells that are associated with apoptosis, the cell cycle, cell differentiation and migration. Indirect and direct co-culturing of U87 and U373 cells showed mutually opposite effects on temozolomide resistance. In conclusion, definition of transcriptional alterations of distinct glioblastoma cells upon co-culturing provides better understanding of the mechanisms of glioblastoma heterogeneity, which will provide the basis for more informed glioma treatment in the future.

## INTRODUCTION

Glioblastoma multiforme (GBM) is the most lethal of all of the brain tumors, because of its single-cell infiltration into normal brain parenchyma and its local recurrence. The median survival of patients with GBM remains low, at 15 to 16 months, despite the development of novel therapeutic modalities [1]. As stated by Stieber et al. [2], to advance personalized treatment and improve clinical outcomes, un understanding of intra-tumor heterogeneity at the cellular and genetic levels is mandatory.

Gene expression signatures were believed to more accurately predict for the outcome of GBM patients than histology alone [3]. Hence GBM were classified based on their gene expression patterns, somatic mutations, and DNA copy numbers as the proneural, neural, classical and mesenchymal subtypes [4]. However, these subtypes and further (sub) classifications according to The Cancer Genome Atlas Research Network - TCGA that have arisen through next generation sequencing analyses [5, 6] have still failed to sufficiently stratify patients and to successfully predict their responses to therapy, due to intra-tumor heterogeneity [7, 8].

Similar to other tumors, GBM contain heterogeneous regions of cells that are highly proliferative or invasive, with apoptotic and necrotic areas, as well as regions of intensive angiogenesis [9]. This is due to the clonal diversity within GBM, as has been confirmed by gene expression profiling [10], FISH analysis, and comparative genomic hybridization [11, 2]. According to ploidy, GBM were recently sub-classified as mono-genomic or poly-genomic tumors, with the former consisting of a pseudo-diploid tumor cell clone admixed with normal stromal cells, and the latter consisting of multiple tumor cell clones mixed with pseudo-diploid cells [2]. Moreover, epigenetic changes such as the O6-methylguanine DNA methyltransferase (*Mgmt*) gene methylation patterning, have been shown to modulate responses to temozolomide in GBM [12]. It is thus clear that the overall combined “omics” results from the analysis of this tumor tissue cannot reliably explain the complex cellular processes within the GBM tumor mass [13].

Different GBM subtypes might reflect different histological origins of GBM stem-like cells (GSCs) [14]. Genetically divergent GBM cell populations might have evolved from GSCs of different origins that expressed variable patterns of stem-cell markers, such as CD133, CD15, A2B5, and CD44, due to which they might harbor different tumorigenic potential [2]. The heterogeneity of a GBM might also arise from differences in differentiation of the progenitors into the tumor cells under the pressure of the tumor microenvironment, containing endothelial, immune, and normal stem cells of mesenchymal and hematopoietic origins [3]. Different tumor cell populations might thus not be just inert bystanders; rather their interactions with the tumor microenvironment and among themselves might have an impact on the tumor growth and its drug resistance.

Increasing numbers of studies are addressing the intra-tumor heterogeneity, through investigations into the interactions among various cancer cells. However, they rarely address this at the molecular interactome level, which might provide the best information regarding the impact of tumor heterogeneity on tumor growth and invasion, and on the prediction of treatment response. Here, we used DNA microarrays to study the interactions between genotypically and phenotypically distinct GBM cell lines, and to investigate their impact on cellular processes in direct and indirect co-cultures *in vitro*. As the *in vitro* cellular models, we selected phenotypically distinct cell lines that are often used as cell models to study GBM: the rapidly proliferating U87 GBM cells; and slowly proliferating U251 and U373 GBM cells. We report that the U87 and U373 cells differ significantly in their gene expression fingerprints and express phenotypes that resemble the neuronal and mesenchymal characters, respectively. Similarly, neuronal and mesenchymal phenotypes were ascribed to GSCs by Denysenko [8]. Here, we are also reporting on cellular processes, such as cell proliferation, colony forming, invasion, and chromosomal instability, and on the resistance of these cells to the alkylating agent temozolomide (TMZ), which was dysregulated in these co-cultured GBM cells. We have associated these processes with their respective transcriptomic changes in indirect co-cultures. To our knowledge, this is the first in-depth analysis of interactions between distinct GBM cell lines, and we show that GBM clones within a tumor mass do not just co-exist, but rather they cooperate with each other.

## RESULTS

### Established GBM cell lines show different growth dynamics, cytokine expression and morphology

**Figure 1:**
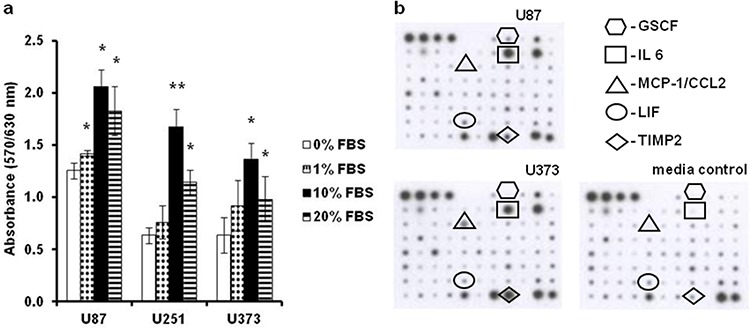
The U87, U251 and U373 GBM derived cell lines differ in their serum dependence and cytokine secretion **a.** Cells of all three cell lines were grown in growth media with increasing FBS concentration (as indicated), and their proliferation indices were determined after 72 h using the MTT assay. **b.** Representative cytokine macroarray profiling of the media conditioned by U87 and U373 cells. Each dot on the membranes represents detection of a specific chemokine (as indicated).

**Figure 2:**
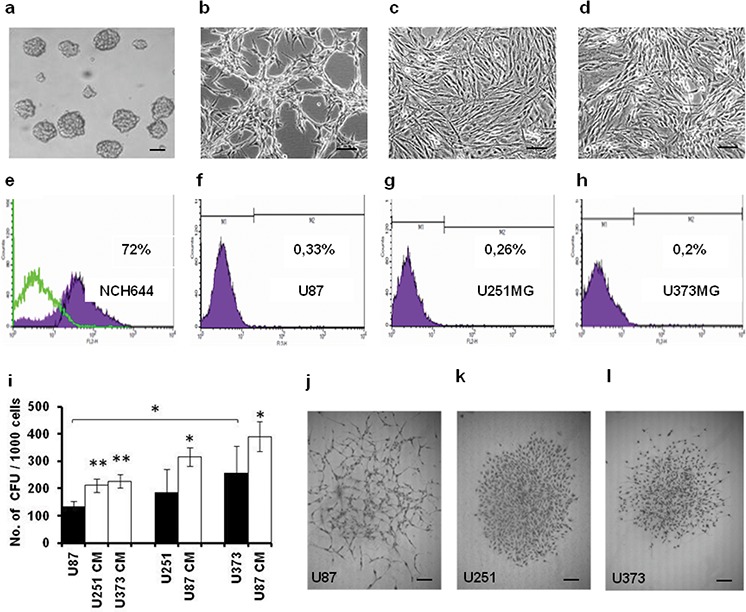
The U87, U251 and U373 GBM-derived cell lines have different morphologies, CD133+ GSC levels, and CFU formation **a-d.** Representative images of morphology of NCH644 (a), U87 (b), U251 (c) and U373 (d) cells under 100× magnification (scale bars 100 μm). **e-h.** Expression of CD133/AC133 anti-gene (CD133/2 epitope) in these cells (as indicated) evaluated by flow cytometry. **i.** Quantification of CFU formed by U87, U251 and U373 cells (as indicated) grown in growth medium and CM. **j-l.** Representative images of morphology of U87, U251 and U373 colonies (as indicated) under 40× magnification (scale bars 50 μm). Error bars represent SEM. *p < 0.05, **p < *0.01*.

U87, U251 and U373 GBM cells were initially assayed for their proliferation under increased serum conditions (Figure 1a), and for their cytokine expression (Figure 1b). U87 cells showed superior growth to U251 and U373 cells, as they were more proliferative, when grown under serum-deprived, normal (10% fetal bovine serum [FBS]), and serum enriched conditions (Figure 1a). High serum (i.e. 20%) inhibited the growth of all three of these cell lines. Of the 79 cytokines measured, granulocyte colony stimulating factor (GSCF), interleukin 6 (IL6), chemokine ligand 2 (CCL2), leukemia inhibitory factor (LIF) and tissue inhibitor of metalloproteinases (TIMP) appeared to be differentially secreted from U87 and U373 cells (Figure 1b). Consistent with their proliferative and secretory differences, different morphologies of these GBM cell lines were noted (Figure 2b–2d). The rapidly growing U87 cells appear morphologically distinct (Figure 2b) from the slowly growing U251 and U373 cells (Figure 2c, 2d). Both U251 and U373 cells had a mesenchymal-like morphology, whereas U87 cells with their long thin protrusions resembled a neuronal morphology.

### Established cell lines differ in their stem-like cell characteristics

Different glioma clones isolated from the same GBM have previously been shown to have different tumorigenic potential that correlated with their GSC content. We therefore determined the cell-surface expression of the prominin 1/CD133 antigen in U87, U251 and U373 cells, as compared to the positive control GSC spheroids of the NCH644 cell line (Figure 2a–2h). The percentage of CD133+ cells varied across all three of these GBM cell lines, with U87 cells showing the highest levels (0.33%) and U373 the lowest levels (0.20%) of CD133+ cells (Figure 2e–2h). The potential for self-renewal of these tumor cells was measured according to their numbers of colony forming units (CFU), whereby U87 cells showed lower CFU compared to U373 cells (Figure 2i). As GBM cells use paracrine signals for their cellular cross-talk, the impact of U87 cell conditioned media (CM) on the CFU of U373 and U251 cells, and *vice versa*, were analyzed. CFU formation of all of these cell lines was increased in cultures with CM (Figure 2i). U87 cells exposed to U373 CM showed an almost doubling of their CFU, whereas with the slow-growing U373 cells there was only a 30% increase in CFU upon exposure to U87 CM. The presumed neuronal and mesenchymal characters of these cells mirrored in the appearance of their colonies, with U87 cells forming relatively loose colonies, compared to the U251 and U373 cells, which both formed compact and evenly rounded colonies (Figure 2j–2l).

### Proliferation of GBM cells changes in the co-cultures

**Figure 3:**
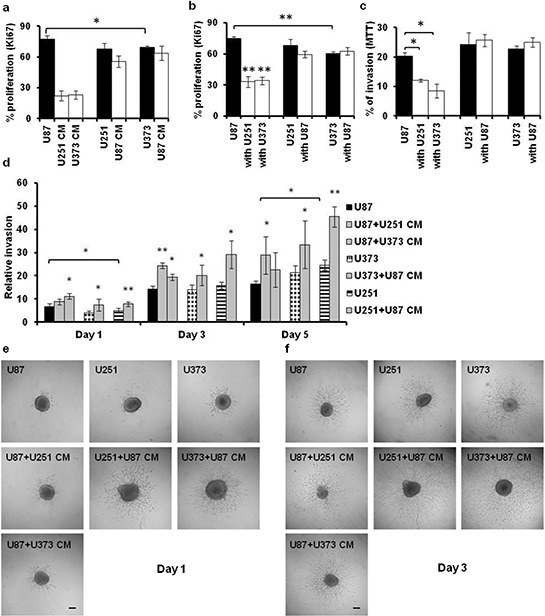
Proliferation and invasion of the U87, U251 and U373 GBM-derived cell lines in indirect co-cultures and CM cultures **a,b.** Proliferation of GBM cells (as indicated) after 72 h, determined using Ki-67 immuno-staining in CM cultures (a) and indirect co-cultures with the indicated GBM cells (b). **c, d.** Invasion of GBM cells determined using the 2D-Boyden chamber assay after 72 h (c), and using the 3D-spheroid/collagen embedding assay after 1-, 3- and 5- days (d). **e, f.** Representative images of U87, U251 and U373 spheroid invasion in collagen after 1 (e) and 3 (f) days (as indicated) under the 40× magnification (scale bars, 50 μm). Error bars represent SEM. *p < 0.05, **p < *0.01*.

The proliferation potential of GBM cells was measured by Ki67 immuno-staining, which demonstrated that U87 cells proliferated significantly faster than U373 cells (Figure 3a, 3b), which was consistent with the results of the 3-(4,5-dimethylthiazol-2-yl)-2,5-diphenyltetrazolium bromide (MTT) assays (Figure 1a). The proliferation of U87 cells was decreased by > 50%, when they were cultured either with the U251/U373 CM or were indirectly co-cultured with the slow-growing GBM cells (i.e. U251, U373 cells) (Figure 3a, 3b). However, for 251 and U373 cells, neither their growth in U87 CM nor their indirect co-culturing with U87 cells evoked any changes in their cell proliferation.

### Invasion potential of GBM cells changes in the co-cultures

The impact of paracrine mediators on cell invasion was tested by using the 2D-Boyden chamber assay (indirect co-cultures, CM cultures) and the 3D-spheroid collagen embedding assay (CM cultures). There was decreased cell invasion of U87 cells in the indirect co-cultures with U251 and U373 cells, whereas again there were no changes in U251 and U373 cell invasion (Figure 3c). The same was observed in the 2D-Boyden chamber CM cultures (data not shown). Contrary to the indirect co-cultures and the CM cultures in the 2D set up of the Boyden chambers, invasion of all of the cell lines in the 3D set-up (collagen embedded spheroids exposed to CM) was increased (compare Figure 3c and Figure 3d). This might first be due to the different matrices used, i.e. matrigel *versus* collagen in the 2D and the 3D set ups, respectively, which might have affected both the adhesion and invasion of the cells. Secondly, the multi-cellular structure of the 3D spheroids (with the mediators secreted by the inner cells), might have influenced U87 cells differently, and enabled them to override the effects of the paracrine mediators present in the CM of U251 and U373 cells. As differences in the relative invasion were observed between the control U87 and U373 cells, with the relative invasion of the control U87 cells higher on day 1 and lower on day 5 (Figure 3d–3f) when compared to the control U373 cells, this would imply on direct homotypic cell interactions within the U87 spheroids to indeed have an early enhancing and a later inhibiting effect on U87 cell invasion.

### Genomic stability of GBM cell lines correlates with their growth

**Figure 4:**
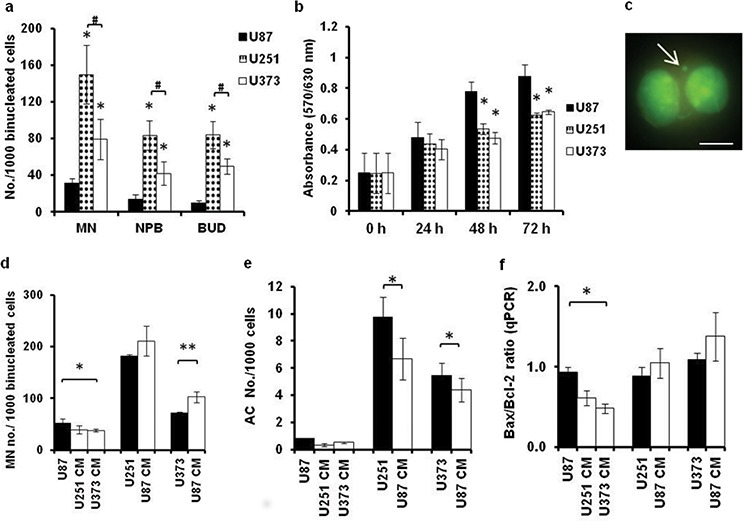
Media conditioned by GBM cells differentially affect genomic stability of U87, U251 and U373 cells **a, b.** Micronuclei (MN), nucleoplasmatic bridges(NPB) and nuclear buds (BUD) detected according to cytokinesis-block micronucleus cytome assay for binucleated U87, U251 and U373 cells (a) and according to proliferation detected by the MTT assay at 24, 48 and 72 h (b). **c.** Representative image of MN in a binucleated U87 cell stained with acridin orange and observed under 400× (fluorescence microscopy, scale bar 50 μm). **d-f.** Quantification of the MN (d), apoptotic/necrotic cell (AC) numbers determined using acridin orange staining (e) and the Bax/Bcl-2 ratio determined using qRT-PCR (f) in U87, U251 and U373 cells grown normally or exposed to the indicated CM for 72 h. Error bars represent SEM. *p < 0.05, **p < *0.01*.

To determine whether the genomic instability that can lead to the evolution of aneuploid cells is affected by the paracrine GBM cell-cell interactions, we performed the cytokinesis-block micronucleus cytome assay with U87, U251, and U373 cells exposed to CM (Supplementary Figure S1). The results of the MTT assay (used for estimation of cellular proliferation) in all of the GBM cell lines were inversely correlated with their levels of genomic instability, as evaluated by the number of micronuclei (MN), nucleoplasmic bridges (NPBs) and nucleated buds (BUDs) in bi-nucleated cells (Figure 4a) of the normally proliferating cells (Figure 4b–4c). U87 cells showed lower numbers of MN, NPBs, and BUDs compared to both of the slow-growing U251 and U373 cell lines. Between these latter two cell lines, U251 cells showed the highest numbers of MN, NPBs and BUDs (Figure 4a).

When exposing these GBM cells to the CM of the other cell type(s), the number of MN in U87 cells decreased with exposure to U373 CM, whereas the number of MN in U373 cells increased with exposure to U87 CM (Figure 4d). This decrease in the chromosomal instability in U87 cells correlated with the demonstrated inhibition of proliferation of these U87 cells by U373 CM (compare Figure 3a with Figure 4d). U87 CM also increased the number of apoptotic cells in both U251 and U373 cells, whereas no such effect was noted in U87 cells exposed to U251 CM or U373 CM (Figure 4e). This implies that U87 cells have a higher apoptotic threshold compared to U251 and U373 cells. Consistent with this, the Bax (Bcl-2 associated X protein)/Bcl2 (B-cell CLL/lymphoma 2) ratio was decreased in U87 cells exposed to U373 CM (Figure 4f). Conversely, and although not significant, there was an increase in the Bax/Bcl2 ratio in U251 and U373 cells exposed to U87 CM. Together, these data imply that U251 CM and U373 CM act towards increased genomic stability of U87 cells, whereas U87 CM might have opposite effects; i.e., it might decrease genomic stability of U251 and U373 cells, and enhance their apoptotic tendency.

### Sensitivity of GBM cells to temozolomide changes between indirect and direct co-cultures

**Figure 5:**
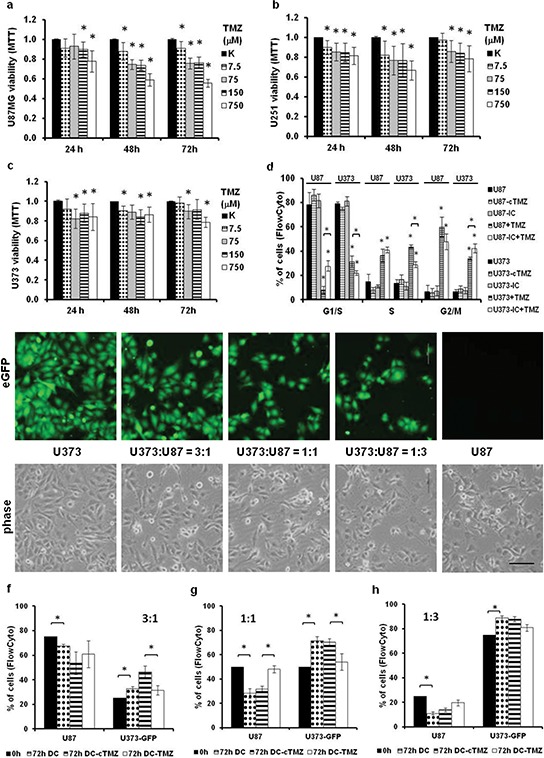
Sensitivity of GBM cells to temozolomide (TMZ) in co-cultures according to cell-cycle deregulation **a-c.** Viability of U87 (a), U251 (b) and U373 (c) cells after TMZ treatment for 24, 48 and 72 h, as determined with the MTT assay. **d.** Cell-cycle analysis of monocultured and indirectly co-cultured (IC) U87 and U373 cells in growth medium and treated with 750 μM TMZ for 72 h, according to propidium iodide staining and flow cytometry. **e.** Representative fluorescent (upper panels) and phase contrast (lower panels) images of monocultured U87 and U373-eGFP cells and their co-cultures plated at different cell ratios (3:1, 1:1; 1:3) as observed under 200× magnification (scale bar 100 μm). **f-h.** Relative numbers of U87 and U373-eGFP cells in 3:1 (f), 1:1 (g), 1:3 (h) direct co-cultures as detected by flow-cytometry after 72 h of TMZ treatment. DC, untreated direct co-culture; DC-cTMZ, control 0.5% DMSO treated direct co-culture; DC-TMZ, direct co-culture treated with 750 μM TMZ. Error bars represent SEM. *p < 0.05, **p < *0.01*.

Different clones within a GBM might acquire different resistances against chemotherapy. Apart from mutations known to change cellular resistance, our aim was to measure the effects of co-culturing different GBM cells on their resistance to TMZ over 72 h. The sensitivities of U87, U251, and U373 cells to TMZ were analyzed by measuring cell viability with the MTT assay (Figure 5a–5c). The highest viability decrease (20%) was observed for U87 cells at the highest TMZ concentrations. Lower viability decreases for U251 and U373 cells exposed to TMZ are consistent with their slow growth and imply their higher intrinsic resistance to TMZ, compared to U87 cells (compare Figure 5a with Figure 5b–5c). That is consistent with the cell-cycle analyses of U87 and U373 cells exposed to TMZ (Figure 5d, compare the 4^th^ columns of each dataset), where fewer U87 cells in G1/S phase were noted, when compared to U373 cells.

Moreover, in the indirect co-cultures of U87 and U373 cells after TMZ treatment, more U87 cells remained in G1/S phase, when compared to treated mono-cultured U87 cells. In co-cultured U373 cells, the inverse response was noted (Figure 5d, compare 4^th^ and 5^th^ columns of each dataset for both cell lines). This suggested that in the indirect co-cultures of U87 and U373 cells, the resistance of U87 cells to TMZ increased, whereas it decreased for U373 cells.

To determine whether these co-culture effects might influence the cellular composition of the direct co-cultures upon TMZ treatment, direct co-cultures of U87 and U373-eGFP cells were set up at different ratios (Figure 5e). These were then evaluated by flow cytometry (Figure 5f–5h) after 72 h exposure to TMZ, and for the control (dimethylsulfoxide; DMSO). U87 cells decreased their proliferation rates in direct co-cultures with U373-eGFP cells at all of the ratios tested. Conversely, U373-eGFP cells increased their proliferation rates in direct co-cultures with U87 cells, which is consistent with the previous proliferation measures (compare Figure 5 with Figure 3a–3b). As in the cell cycle analysis, TMZ increased the proliferation rates of U87 cells in direct co-culture, whereas it decreased the proliferation rates of U373 cells, when compared to non-treated directly co-cultured cells (Figure 5f–5h). This was most evident for 1:1 direct co-culture (Figure 5g), which again implies that U87 cells sensitize U373 cells to TMZ, and U373 cells protect U87 cells from TMZ toxicity, a concept that was also observed in the indirect co-cultures (Figure 5d).

Taken together, we have demonstrated that this cellular cross-talk has an impact on the resistance of co-cultured cells *in vitro*, and we speculate that similar interactions between different clones that co-exist in a GBM *in vivo* might likewise influence the overall tumor therapy response for alkylating agents.

### Transcriptomic analysis supports neuronal and mesenchymal phenotypes of U87 and U373 cells, respectively

**Figure 6:**
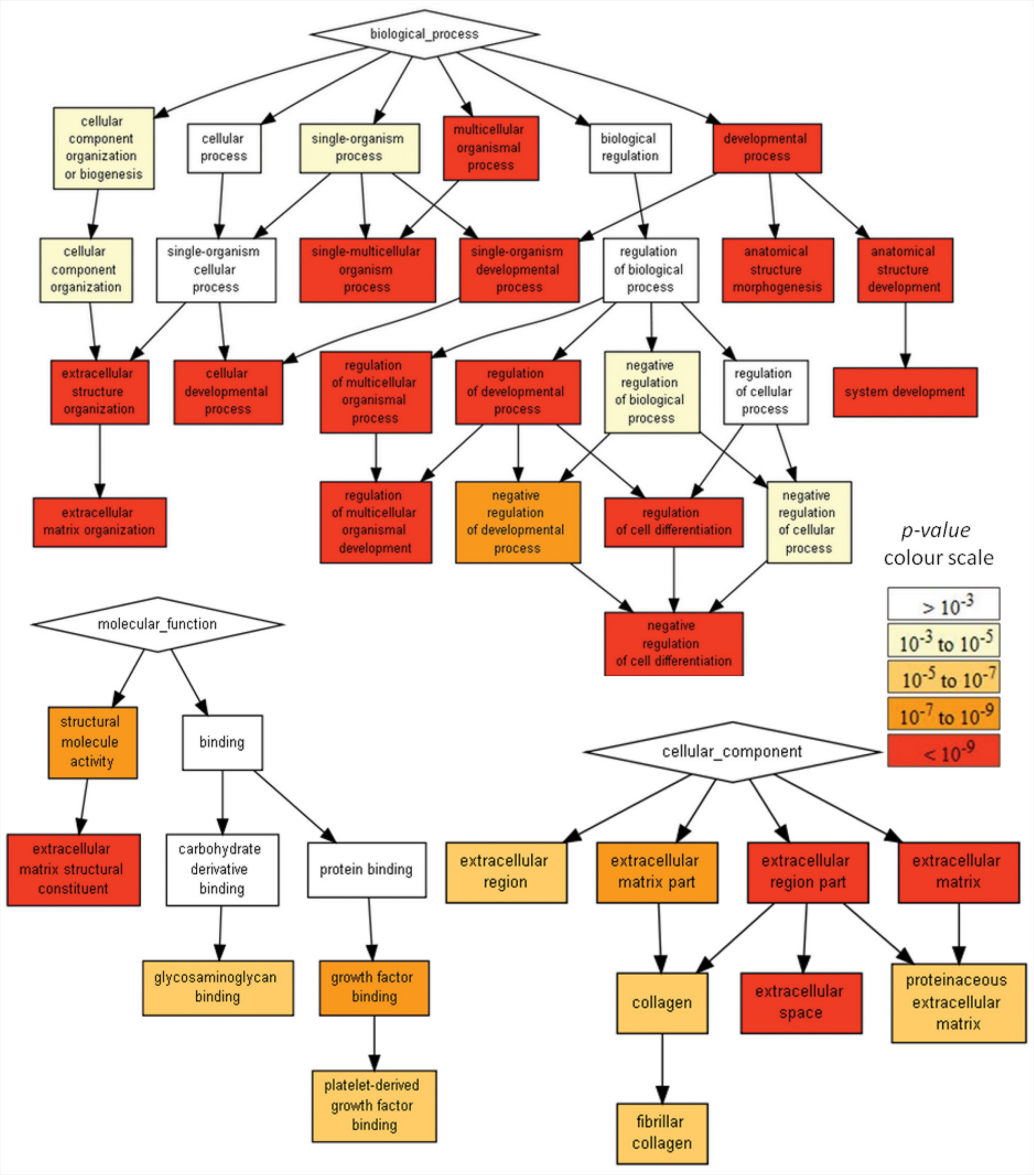
Gene Ontology enRIchement anaLysis and visuaLisAtion (Gorilla) reveals the GO terms in Process, Molecular Function and Cellular Component gene ontology domains, enriched with genes differentially expressed in U87 cells when compared to U373 cells The pathways analysis was conducted at http://cbl-gorilla.cs.technion.ac.il/ on 8948 differentially expressed genes (*p* < 0.01). Only the GO terms with *p*-value < 10^−9^ for Process, and *p*-value < 10^−5^ for Molecular Functions and Cellular Components are presented. According to gene expression and profiling studies, at least two subtypes of GBMs can be distinguished; one with a prevailed gene expression pattern associated with neural development and the second with a prevailing gene expression pattern associated with extracellular matrix components. The latter is shown also by U373 cell gene expression, when compared to U87 cell gene expression.

Although both U87 cells and U373 cells were derived from primary human GBM, they differ in their gene expression patterns (Supplementary Table S1, *p*-value < 0.01). Comparative gene expression analysis [15] of U87 and U373 cells revealed that they differ in the expression of 5275genes (absolute logFC > 1, *adj. *p*-value* < 0.01). U373 cells showed increased expression of surface markers associated with mesenchymal stem cells, i.e. CD29 (integrin b1/fibronectin receptor), CD90 (Thy-1 cell surface antigen) and CD105 (endoglin, TGF-b receptor), when compared to U87 cells. The Gene Ontology (GO) term enrichment and visualization analysis using Gorilla [16] (Figure 6) further revealed enriched GO terms in the Process (regulation of cell differentiation, extracellular matrix organization), Molecular Function (transmembrane receptor signaling receptor activity) and Cellular Component (extracellular space, clathrin-coated endocytotic vesicle membrane) domains as further features in which U373 cells differ from U87 cells. These appear consistent with the neuronal and mesenchymal phenotypes, that were initially ascribed to these U87 and U373 cells, respectively, in the above analyses.

### Alterations in gene expression of co-cultured U87 and U373 cells support their phenotype changes

Comparative transcriptomics analyses revealed differences between monocultured and indirectly co-cultured U87 and U373 cells in terms of their differentially expressed genes (Supplementary Tables S2, S3). U87 cells grown in indirect co-culture with U373 cells showed significantly changed expression of 264 genes (*p*-value < 0.01), compared to U373 cells, where 221 genes were differentially expressed (*p*-value < 0.01) in co-culture. This indicates that both cell types have an effect on each other when in the co-culture. This became more evident when high stringency criteria were applied (FC > 1, *adj. *p*-value* < 0.01) [15], which generated only 21 differentially expressed genes in co-cultured U87 cells (Table 1), and 60 differentially expressed genes in co-cultured U373 cells (Table 2). The deregulation of *Ccl20* (Chemokine C-C motif ligand 20), *Dpp4* (Dipeptidyl-peptidase 4) and *Egflam* (EGF-like, fibronectin type III and laminin G domains) gene expression in co-cultured U87 cells observed in the cDNA microarrays was validated by qRT-PCR. In U87 cells, it confirmed two-fold increases in *Ccl20* and *Dpp4* expression, and two-fold decrease in *Egflam* expression, whereas their expression did not change significantly in co-cultured U373 cells (Supplementary Figure S2.a–S2.c). The qRT-PCR results for *Ccl20, Dpp4* and *Eglfam* in co-cultured U87 cells were in agreement with the log_2_FC obtained from the cDNA microarray data analysis. Moreover, the most deregulated genes in indirectly co-cultured U87 cells included those involved in proliferation and inflammation (*Gpr86, Dact1*, Rgs2, *Dpp4, Fap, Edn1, Il1A*), migration and adhesion (*Csf2, F3, Ccl20, C1QTnf5, Cpa6, Egflam, Plod 2, Fgd3, Hhex*). In contrast in co-cultured U373 cells, the genes that were identified as differentially expressed were mainly associated with apoptosis and the cell cycle (*Fosl1, Txnip, Btg2, Rgcc, Efhd1, Ier3, Tnaip3, Myc*), or cell differentiation (*Csf2, Gdf15, Il1B, Maff, Dlx5, Krt8*) and migration (*Il8, Cxcl2, Ccl2, Ca12, Fgr, Gper, Eps8L1*). These co-culture gene expression data are in line with the observed differences in cellular responses relating to the proliferative and invasive behaviors in the co-cultured cells, where down-regulation of migration and adhesion genes is consistent with the decreased invasion observed in U87 cells exposed to U373 CM. Likewise, increased expression of differentiation genes with decreased expression of cell-cycle-related genes in U373 cells indirectly co-cultured with U87 cells would explain their decreased proliferative index, as well as their temozolomide resistance.

**Table 1:** Differentially expressed genes of co-cultured U87 cells

Gene symbol	Gene name	Entrez ID	log_2_FC	*p*-value	Gene function
GPR84	G protein-coupled receptor 84	53831	1.58	0.0002	inflammation, prolifeation
PTGS2	prostaglandin-endoperoxide synthase 2	5743	1.36	0.0031	inflammation, mitogenesis, apopotosis
CCL20	chemokine (C-C motif) ligand 20	6364	1.36	0.0044	chemotaxix, chemokinesis
DPP4	dipeptidyl-peptidase 4	1803	1.21	0.0019	immune response (T-cell)
SLC16A6	solute carrier family 16, member 6	9120	1.18	0.0016	regulation of proton transport
CSF2	colony stimulating factor 2 (granulocyte-macrophage)	1437	1.18	0.0009	cell differentiation, migration/adhesion
C6orf204	chromosome 6 open reading frame 204	387119	1.14	0.0038	neoplasm formation
APOH	apolipoprotein H (beta-2-glycoprotein I)	350	1.10	0.0059	migration, apoptosis, angiogenesis
LTK	leukocyte receptor tyrosine kinase	4058	1.10	0.0019	cell transformation, reprogramming
EDN1	endothelin 1	1906	1.09	0.0009	endothelial dysfunction, inflammation
F3	coagulation factor III (thromboplastin, tissue factor)	2152	1.08	0.0025	migration, coagulation, angiogenesis
BMF	Bcl2 modifying factor	90427	1.07	0.0026	apoptosis
KBTBD7	kelch repeat and BTB (POZ) domain containing 7	84078	1.05	0.0002	transcriptional signalling
DACT1	dapper, antagonist of beta-catenin, homolog 1	51339	1.04	0.0011	embryo /neural tube development
ZNF226	zinc finger protein 226	7769	1.03	0.0013	miscelaneous
C1QTNF5	C1q and tumor necrosis factor related protein 5	114902	1.02	0.0005	adhesion, extracell. deposit formation
IL1A	interleukin 1, alpha	3552	1.00	0.0039	inflammation, hematopoesis, apooptosis
CPA6	carboxypeptidase A6	57094	−1.02	0.0011	proteolysis, extracell. neuropeptides sig.
EGFLAM	EGF-like, fibronectin type III and laminin G domains	133584	−1.04	0.0001	matrix organisation, cell adhesion
HHEX	hematopoietically expressed homeobox	3087	−1.06	0.0031	cell differentiation, apoptosis
FGD3	FYVE, RhoGEF and PH domain containing 3	89846	−1.33	0.0000	morphology & motility regulation

* The differentially expressed genes in U87 cells grown in co-culture with U373 cells relative to U87 cells grown in monoculture, after passing the high-stringency criteria of *p*-value < *0.01* and absolute log_2_FC > 1.0. The up-regulated genes are followed by the down-regulated genes.

**Table 2:** Differentially expressed genes of co-cultured U373 cells

Gene symbol	Gene name	Entrez ID	log_2_FC	*p*-value	Gene function
IL8	interleukin 8	3576	5.17	3.31E-06	chemotaxis, migration, angiogenesis
CSF2	colony stimulating factor 2 (granulocyte-macrophage)	1437	4.74	2.63E-05	differentiation, survival, migration
CXCL2	chemokine (C-X-C motif) ligand 2	2920	3.03	5.00E-05	adhesion, migration/invasion, chemotaxis
GDF15	growth differentiation factor 15	9518	2.71	8.81E-03	tissue differentiation, homeostasis
IL1A	interleukin 1, alpha	3552	2.51	6.35E-03	hematopoiesis, apoptosis
IL1B	interleukin 1, beta	3553	2.24	6.38E-04	inflammation, proliferation, differentiation
CCL2	chemokine (C-C motif) ligand 2	6347	2.16	6.71E-06	immunoregulation, invasion
IER3	immediate early response 3	8870	2.14	7.21E-04	survival, DNA repair, apoptosis
CA12	carbonic anhydrase XII	771	1.89	2.04E-03	respiration, migration/invasion, metabolism
MAFF	v-maf musculoaponeurotic fibrosarc. oncog. homolog F	23764	1.71	1.50E-03	differentiation, stress response, coagulation
MAP3K8	mitogen-activated protein kinase kinase kinase 8	1326	1.59	3.37E-03	lymphocyte activation, IC signal transduction
TNFAIP3	tumor necrosis factor, alpha-induced protein 3	7128	1.55	5.96E-04	immune response regulation, apoptosis
VCAM1	vascular cell adhesion molecule 1	7412	1.46	8.05E-04	cell adhesion, signal transduction
IL6	interleukin 6 (interferon, beta 2)	3569	1.46	5.50E-03	immune cell maturation, immune response
ASNS	asparagine synthetase (glutamine-hydrolyzing)	440	1.38	5.78E-03	cell response, cell-cycle regulation
MYC	v-myc myelocytomatosis viral oncogene homolog	4609	1.36	8.22E-03	cell cycle, apoptosis, transformation
PAPPA	pregnancy-associated plasma protein A, pappalysin 1	5069	1.29	1.79E-03	wound healing, bone remodelling, proteolysis
BIRC3	baculoviral IAP repeat containing 3	330	1.26	1.06E-04	apoptosis/necrosis, spermatogenesis
TGFA	transforming growth factor, alpha	7039	1.26	1.30E-03	profliferation, differentiation, angiogenesis
ABCA1	ATP-binding cassette, sub-family A (ABC1), member 1	19	1.24	7.90E-03	cellular transport, lipid metabolic processes
G0S2	G0/G1switch 2	50486	1.24	3.97E-03	apoptosis, lipid metabolism
IL12A	interleukin 12A (natural killer cell stimulatory factor 1)	3592	1.22	4.57E-03	immune response, migration, differentiation
ABCA9	ATP-binding cassette, sub-family A (ABC1), member 9	10350	1.21	4.11E-04	immune cells' differentiation
SLC6A12	solute carrier family 6, member 12	6539	1.21	1.21E-03	synaptic transmission, cellular transport
FUT11	fucosyltransferase 11 (alpha (1,3) fucosyltransferase)	170384	1.18	3.02E-04	glycosphingolipid metabolism
IL4I1	interleukin 4 induced 1	259307	1.18	3.57E-03	inflammation, immune cell proliferation
NFKBIZ	NF kappa light polyp. gene enhancer in B-cells, zeta	64332	1.18	2.67E-03	inflammation, transcription
FLVCR1	feline leukemia virus subgroup C cellular receptor 1	28982	1.17	1.72E-04	erithropoiesis, cell death, transmem.transport
FOSL1	FOS-like antigen 1	8061	1.16	1.32E-03	proliferation, differentiation, transformation
SPRY4	sprouty homolog 4 (Drosophila)	81848	1.16	3.04E-03	apoptosis, proliferation, migration
RELB	v-rel reticuloendotheliosis viral oncogene homolog B	5971	1.14	1.16E-03	transcription, differentiation
DUSP6	dual specificity phosphatase 6	1848	1.14	4.54E-03	proliferation, differentiation, apoptosis
JDP2	Jun dimerization protein 2	122953	1.12	6.26E-04	senescence, transformation
PDGFA	platelet-derived growth factor alpha polypeptide	5154	1.09	7.59E-03	proliferation, motility, embryo development
DKK1	dickkopf homolog 1 (Xenopus laevis)	22943	1.09	1.04E-03	embryo development, invasion
ETV4	ets variant 4	2118	1.06	8.77E-04	proliferation, differntiation, tissue morphogenesis
HMGA1	high mobility group AT-hook 1	3159	1.06	9.75E-04	transcription, cell-cycle, motility, survival
PTPRE	protein tyrosine phosphatase, receptor type, E	5791	1.06	3.68E-03	cytokine signal transduction
CD47	CD47 molecule	961	1.05	7.07E-03	adhesion, survival, autophagy, ECM organisation
SFPQ	splicing factor proline/glutamine-rich	6421	1.04	1.36E-03	proliferation, mRNA processing, DNA repair
ALOX5AP	arachidonate 5-lipoxygenase-activating protein	241	−1.03	5.41E-04	lipid metabolism, inflammation
PRKCZ	protein kinase C, zeta	5590	−1.04	6.23E-03	differentiation, metabolic reprogramming
CBLN2	cerebellin 2 precursor	147381	−1.06	5.12E-03	synaptic signal transmission, synapse assembly
ALAD	aminolevulinate dehydratase	210	−1.06	8.31E-04	heme biosynthesis, neural sensistivity
EPS8L1	EPS8-like 1	54869	−1.07	2.56E-03	actin based motility, actin reorganization
SERTAD4	SERTA domain containing 4	56256	−1.10	2.44E-03	transcription regulation
KRT8	keratin 8	3856	−1.11	2.84E-03	differentiation, invasion
CORO6	coronin 6	84940	−1.14	2.05E-03	actin cytoskeleton organization
GLDN	gliomedin	342035	−1.19	4.51E-03	assembly of Ranvier's nodes, SC-axon interaction
VAV3	vav 3 guanine nucleotide exchange factor	10451	−1.20	2.14E-04	resistance, survival, growth, neoangiogenesis
GPER	G protein-coupled estrogen receptor 1	2852	−1.20	4.56E-03	migration/invasion, apoptosis, cell-cycle
KRT13	keratin 13	3860	−1.23	4.13E-04	differentiation, tissue morphogenesis
CDO1	cysteine dioxygenase, type I	1036	−1.26	3.62E-03	growth metabolism
EFHD1	EF-hand domain family, member D1	80303	−1.27	1.21E-03	apoptosis, diff., neuron development, stress r.
DLX5	distal-less homeobox 5	1749	−1.30	8.25E-04	differentiation, limb development, proliferation
FGR	Gardner-Rasheed feline sarcoma viral oncog. homolog	2268	−1.35	4.21E-04	migration, adhesion, cell shape regulation
RGCC	regulator of cell cycle	28984	−1.39	9.52E-04	cell-cycle progression, ECM-transition
BTG2	BTG family, member 2	7832	−1.52	8.29E-03	cell-cycle, ECM-transition, migration, invad. f.
BMF	Bcl2 modifying factor	90427	−1.57	3.80E-03	apoptosis
TXNIP	thioredoxin interacting protein	10628	−2.93	1.24E-05	apoptosis, stress fiber formation, autophagy

* The differentially expressed genes in U373 cells grown in co-culture with U87 cells relative to U373 cells grown in monoculture, after passing the high-stringency criteria of *p*-value < *0.01* and absolute log_2_FC > 1.0. The up-regulated genes are followed by the down-regulated genes.

## DISCUSSION

Knowledge of intra-tumor heterogeneity at the genetic, cellular and functional levels is crucial for an understanding of GBM resistance to treatment, and thus for improvements in GBM therapies [2, 17]. Although Steiber et al. [2] confirmed the existence of GBM cell heterogeneity *in vivo*, through a systematic study of the transcriptomic and genomic profiles of sorted primary GBM cells, to the best of our knowledge, to date, the intra-tumoral interactions among different GBM clones have not been studied at the molecular level. Here, we used the established U87, U251, and U373 GBM cell lines as our *in vitro* cellular models, to identify de-regulated genes and the underlying phenotypic changes observed in indirectly and directly co-cultured U87 and U373 cells, including their responses to frequently used alkylating drug TMZ. We are aware that established cell lines differ in their gene expression signatures and ploidy from the primary GBM clones *in vivo* [2], and as such might not be their best representatives. However we agree with Li et al. [18], who showed that there are discrete features of GBM tumors that are conserved in the established cell lines, and that by defining the divergence of the established cell lines from the original tumor type, they concluded that the established GBM cell lines can nonetheless serve as clinically relevant glioma models for a given aim and experimental design.

Although, previous transcriptomics analyses classified GBMs into four subtypes, [4, 14, 19], Denysenko and co-workers [8] demonstrated that GSCs, derived from primary GBM, belong either to a readily growing neuronal type of cells with prevailing expression of neuronal development genes [4] or to a slowly proliferating mesenchymal type of cells with gene expression patterns underlying their extracellular matrix synthesis/degradation activity [4]. Recently, a proneural-to-mesenchymal shift in gene expression patterns was shown to occur in subpopulations of GBM cells as a response to radiation [20]. The acquisition of the mesenchymal phenotype not only enhanced cellular resistance to radiation, but also increased the invasiveness of the cells with the mesenchymal phenotype [8, 20, 21]. The known U87 and U373 cells appear to behave as characterized cells of neuronal and mesenchymal types respectively. They differ in their expression of genes associated with developmental processes, cell differentiation and extracellular matrix organization (Figure 6). Such a distinction between two subtypes of GBM had already been suggested [4]. Thus, we believe that the interactions between these U87 and U373 (and similarly for U251) model GBM cell lines might well mirror these two GBM subtypes *in vivo*.

Clonal evolution of tumors is driven by the genomic instability of tumor-initiating cells, which results in a plasticity of GSCs and their malignant progenitors [22] that is also reflected in the established tumor cell lines [17]. Intercellular cross-talk might either accelerate or counteract the genetic stability. We observed that higher genetic instability was reflected in more numerous MN, NPBs and BUDs in U251 and U373 cells *versus* U87 cells. The number of MN increased in U373 cells upon exposure to U87 CM, whereas U251/U373 CM cells had the opposite effect on U87 cells. This is consistent with the anti-proliferative effect that U373 cells have on U87 cells. Similar correlations between increased MN and proliferation were observed in pediatric SF188 GBM cells [23]. Also, paracrine and direct interactions between U87 and U373 cells resulted in decreased proliferation of U87 cells, whereas proliferation of co-cultured U373 cells was not affected. Consistent with this, in co-cultured U87 cells there was up-regulation of the genes *Gpr84* (G-protein coupled receptor 84), *Dact1* (Dishevelled-binding antagonist of beta-catenin 1) and *Rgs2* (Regulator of G-protein signalling 2). Expression of *Gpr84* is known to be enhanced by tumor necrosis factor (TNF) α and IL-1 [24], both of which also increased in co-cultured U373 cells. These two cytokines might up-regulate *Gpr84* in U87 cells and decrease their proliferation, similar to retinal cells [25]. Next, increased expression of *Dact1* is known to inhibit proliferation of breast cancer cells through inhibition of Wnt/β-catenin signaling [26], while increased expression of *Rgs2* is known to suppress growth of MCF7 and HEK293T breast carcinoma cells [27]. In contrast, neither proliferation changes nor deregulation of the above-mentioned genes were noted in co-cultured U373 cells. This demonstrates that some cellular responses might indeed be GBM cell-subtype dependent.

The increased number of cells with apoptotic morphology among U373 cells exposed to U87 CM implied their increased apoptosis. In contrast, decreased numbers of MN and decreased *Bax*/*Bcl-2* ratios in U87 cells exposed to U251/U373 CM indicate a higher apoptotic threshold of U87 cells. Consistent with this, we noted altered expression of anti-apoptotic and pro-apoptotic genes in both U87 and U373 cells. Up-regulation of the *Ptgs2* (Prostaglandin-endoperoxide synthase 2) and *Fap* (Fibroblast activation protein, alpha); and down-regulation of *Hhex* (Hematopoietically expressed homeobox) gene in co-cultured U87 cells might have protected these from apoptosis. Namely, *Ptgs2* inhibits apoptosis in glioma xenografts [28], while *Fap* over-expression increases astrocytoma resistance to FAS-mediated apoptosis [29]. Moreover *Hhex* down-regulation, coinciding with down-regulation of Bcl-2 [30], might explain the higher apoptotic threshold of co-cultured U87 cells. In contrast, expression of the *Ier3* (Immediate early response 3) gene, which enhances apoptosis in radiated glioma cells via NFkB, MAPK/ERK and PI3K/Akt signaling [31], was increased in co-cultured U373 cells.

The different responses of U87 and U373 cells initiated by their paracrine interaction might be explained by their differences in expression of the tumor suppressor gene *p53*. The transcription factor P53 is a known regulator of genes involved in the cell cycle, genetic instability, proliferation, and apoptosis [32]. The wild type *p53* status of U87 cells [33] might underlie the observed decrease in proliferation of U87 cells exposed to U251/U373 CM. Their active P53 might have induced cell-cycle arrest and DNA damage repair, which would be reflected in decreased numbers of MN and increased apoptotic threshold in these exposed U87 cells. On the other hand, the mutated *p53* gene status in U373 cells [33] might underlie the increased genomic instability and apoptosis in U373 cells exposed to U87 CM. The up-regulation of *G0s2* (G0/G1 switch 2) expression seen in co-cultured U373 cells might have increased the apoptotic morphology of co-cultured U373 cells, as when G0s2 interacts with BCL-2, it promotes apoptosis by preventing formation of the BAX/BCL-2 hetero-dimer [34].

These apoptotic threshold alterations might result in changed sensitivity of the GBM cells to TMZ, a DNA alkylating agent that is known to impair GBM cell growth and increase survival of patients from 6 months up to 1 year. However, this only relates to GBM patients with inactive (methylated) MGMT [12], as active MGMT can correct for TMZ-induced DNA damage. The U87, U251, and U373 cell lines are known to have a methylated *MGMT* promoter and have been described as TMZ sensitive [35, 36]. We demonstrated that the neuronal-like U87 cells are more resistant to TMZ than the mesenchymal-like U373 cells after 24 h at low concentrations of TMZ, which parallels the resistance of the same phenotypes of GSCs reported by Denysenko et al. [8]. The TMZ resistance of U87 and U373 cells changed in their (in)direct co-cultures *versus* monocultures, possibly due to their altered proliferation, as proliferative cells are more sensitive to cytotoxic agents [17]. These data suggest that mesenchymal-like U373 cells protect neuronal-like U87 cells from TMZ cytotoxicity, and conversely, U87 cells appear to sensitize U373 cells to TMZ. Similarly, the *in vivo* cross-talk between different co-existing clones within a GBM might underlie the differential tumor therapy responses observed. In agreement with the above, we observed increased expression of the *Ptgs2* and *Egr2* (Early growth response 2) genes in co-cultured U87 cells. These reportedly promote resistance of lung cancer cells to cis-platinum [37] and colon cancer cells to 5′-fluorouracil [38].

Gene expression changes observed in co-cultured U87 and U373 cells resulted in altered invasiveness of these GBM cells. Contrary to increased invasion of U373 cells observed in both the 2D and 3D invasion assays, invasion of U87 cells appeared to be highly dependent on the matrix substrate and spatial set-up. When exposed to U373 CM, U87 cell invasion decreased in the indirect 2D co-cultures using matrigel, whereas it increased in the 3D-collagen-embedded U87 spheroids. As effects of similar matrices on gene expression have been reported previously [39], the decreased migration of U87 cells in indirect co-culture with U373 cells might result from increased expression of the *C1qTnf5* (C1q and tumor necrosis factor related protein 5) gene, which enhances adhesion of retinal epithelial cells [40], and decreased expression of the *Plod2* (Procollagen-lysine, 2-oxoglutarate 5-dioxygenase 2) gene, which inhibits sarcoma cell migration [39]. On the other hand, increased invasion of U87 cells from spheroids in the 3D-collagen-embedding CM cultures might be ascribed to increases in the expression of the *Ccl20* (Chemokine C-C motif ligand 20) and *Csf2* (Colony stimulating factor 2) genes, and a decrease in the expression of the *Fgd3* (FYVE, RhoGEF and PH domain containing 3) gene. Namely, *Ccl20* and *Csf2* are both up-regulated in GBM *in vivo*, and even correlate with shorter patient survival [41, 42]. Knock-down of *Csf2* expression also reduces invasion of GL261 cells *in vivo* [41].

With the respect to GBM cell invasion, which is known to be of mesenchymal type and to involve the formation of cellular protrusions, or invadopodia [43], some of the associated genes were also up-regulated and down-regulated in co-cultured GBM cells. Decreased *Fgd3* gene expression is known to increase migration of HeLa cells [44], and this might account for the increased invasiveness of U87 cells exposed to U373 CM. In U373 cells co-cultured with U87 cells, the expression of *Csf-2, IL8* (Interleukin 8) and *IL6* (interleukin 6) increased, which is known to increase MCF-7 cell migration [45]. The increased invasion of U373 cells might also be due to decreased expression of the *Btg2* (BTG family member 2) and *Vav3* (Vav 3 guanine nucleotide exchange factor) genes, which might account for the pro-invasive morphology of U373 cells, through the release of *Btg2* and *Vav3* inhibitory effects on invadopodium formation [46, 47]. Taken together, we speculate that the cross-talk between U87 and U373 cells is responsible for their increased invasion in the 3D spheroid assay, which is possibly mediated by their paracrine and autocrine signals.

In summary, we have demonstrated that GBM cells of distinct phenotypes and *p53* gene status can indeed significantly impact upon each other's phenotype *in vitro*, as shown by their altered proliferation, invasive behavior, and TMZ resistance. This study also provides evidence that the established U87, U251, and U373 GBM cell lines and their co-cultures provide reliable *in vitro* models, as these resemble the *in vivo* intra-tumor interactions of the heterogeneous tumors that contain mixed cell phenotypes. Thus, these *in vitro* models are particularly useful for evaluation of drug response. The molecular markers that were shown here to be up-regulated in our transcriptomic and cytokine analyses can now be further studied and used in anti-proliferative and anti-invasive therapeutic designs for GBM treatments.

## MATERIALS AND METHODS

### Cell culture and conditioned media collection

The U87, U251 and U373 human GBM cell lines (from ATCC, Manassas, VA, USA) were cultured in Dulbecco's modified Eagle's medium (Sigma USA) with 10% fetal bovine serum (FBS, Sigma, USA), 100 U penicillin, 1000 U streptomycin, 2 mM L-glutamine, Na-pyruvate and non-essential amino acids, and they were plated at a density of 15 000 cells/cm^2^. This growth medium was also used as the control medium in the direct and indirect co-culturing experiments. U373-eGFP cells were cultured in the growth medium with 1 mg/mL of geneticin (Sigma, USA). NCH644 cells were grown in neurobasal (NBE) medium (Gibco, Life Tech Corp., UK) with 2 mM L-glutamine, 100 U penicillin, 1000 U streptomycin (all PAA Laboratories, Austria), B-27 (Gibco, UK), 1 U/ml heparin (Sigma-Aldrich, Germany), 20 ng/ml epidermal growth factor (EGF) in 20 ng/ml β-fibroblast growth factor (Gibco, UK), and seeded at 1 × 10^5^ cells/ml. All of the cell lines underwent DNA fingerprint short tandem repeat analysis to confirm their identity [48, 49, 50].

Conditioned media (CM) were obtained by conditioning the growth media for 24 h with the specified confluent cells immediately prior to passaging. CM were centrifuged at 1,500 × *g* for 10 min and mixed with fresh growth medium at a 1:1 ratio prior to use.

### Colony formation assay

Five hundred cells of each cell type were seeded onto 100-mm dishes in duplicates with control or CM of the relevant cell type added. The medium was changed every 5 days. On day 14, colonies containing > 30 cells were counted following staining with 0.4% Giemsa dye (Sigma, USA). As a control medium, a medium conditioned by the non-tumorigenic, normal bone marrow-derived mesenchymal stem cells (MSCs) was also used. For collecting the MSC-CM, the MSCs (male, 33, 6F4085; Lonza Bioscience, Wakersville Inc.) were grown in the above mentioned growth medium for 24 h, prior to passaging. No significant increases of the CFU ability of the GBM cells were noted when these were exposed to MSC CM (Supplementary Figure S3).

### Serum dependence and MTT cell viability assay

Cells were seeded into 96-well plates (6000 cells/well). After 24 h and a wash with phosphate-buffered-saline (PBS), the growth media containing 0%, 1%, 10% and 20% FBS were added to the wells as five replicates. After 48 h, the MTT reagent [3-(4,5-dimethylthiazol-2-yl)-2,5-diphenyltetrazolium-bromide; Sigma, USA] was added. After 3 h incubation, the formazan crystals were dissolved in dimethyl-sulfoxide (DMSO) (Sigma, USA) and the absorbance was measured as the change in optical density (ΔOD 570/690 nm) using microplate reader (Synergy™ HT, Bio-Tec Instruments Inc., USA). To determine TMZ cytotoxicity with MTT, attached cells were treated with media containing 7.5, 75, 150 and 750 μM TMZ for 24, 48 and 72 h. DMSO was used as the Kt control.

### Detection of cytokine release

Fresh growth medium and media conditioned for 72 h by U87 and U373 cells were used to determine the cytokine expression using RayBio Human Cytokine Antibody Array (Ray Biotech Inc., USA), according to the manufacturer protocol. Cytokine signals were detected by chemiluminescence produced with ECL-Plus Western blotting detection reagents (Amersham Biosciences, UK) and a 1-min exposure to X-ray film.

### CD133 marker detection by flow cytometry

Cells were harvested (1 × 10^6^), washed with PBS and incubated with 10 μL CD133-PE antibody (clone 293C3; Miltenyi Biotec GmbH, USA), which recognizes epitope 2 of the human CD133 antigen (CD133/2) and 20 μl isotype control (PE-IgG2b, BD Biosciences, USA), as indicated by the manufacturer (BD Biosciences, USA). Propidium iodide was added to the cell samples for accurate live-cell analysis. The cells were incubated for 20 min, washed and subjected to fluorescence-activated cell sorting (FACS) analysis (BD FACSCalibur™, BD Biosciences, USA) using the CellQuest Software (BD Biosciences, USA), to determine the percentages of viable CD133+ cells.

### Proliferation assay using an anti-Ki67 antibody

The proliferation of indirectly co-cultured cells was determined using 24-well Transwell inserts (Costar, Cornig Inc, NY) with 0.4 μm pore size. Here, 5000 cells were plated in a 50μl drop on poly-L-lysine coated (20 ng/ml, Sigma) cover slips. The co-cultured cells of the relevant other cell type were plated onto the membrane of the insert at a density of 10000 cells /200 μl and left to attach for 2 h. There were then put above the cover slip into 24-well plates, with 800 μl growth medium/well added. Similarly, CM was added to the cover slips for the CM culture set-up. After 72 h, the cells were fixed with 70% methanol for 30 min, permeabilized with 0.1% Triton-X for 10 min, and left in 4% bovine serum albumin for 15 min. These were incubated with the primary mouse anti-Ki67 antibody (1:100 dilution, Abcam, Cambridge, UK) for 1 h, and then with the secondary anti-mouse Alexa-Fluor 488 labeled antibody (1:1500 dilution, Molecular Probes, Paisley, UK), together with Hoechst (5 μg/ml, Sigma-Aldrich, USA) for 90 min. The cover slips were then mounted with Antifade reagent (Life Technologies, USA), and the ratios between the Ki67-stained nuclei and the total nuclei (Hoechst staining) were scored for each experiment in at least 500 cells.

### Cell-cycle analysis of indirectly co-cultured U87 and U373 cells

The indirect co-cultures were set-up in duplicate using 6-well Transwell inserts (Costar, Corning Incorporated, NY, USA) with 0.4 μm pore size. On the bottom of the well, 10^5^ U87 cells were plated in 2 ml, whereas 10^5^ U373 cells were plated into the insert in 1 ml. Cells were left to adhere before being put together and the growth medium with or without temozolomide (TMZ; 750 μM) added. Upon 72 h, the cells were harvested, fixed with 75% ethanol, washed with PBS and resuspended in 0.5 ml propidium iodide/RNase staining buffer (BD Pharmingen, USA). The cell cycle analysis was performed by FACS analysis (BD FACSCalibur™; BD Biosciences, USA) using the CellQuest software (BD Biosciences, USA). The WinMDI and Cyclored software were used to analyze the distributions of the cell populations across the different phases of the cell cycle.

### Proliferation of directly co-cultured U87 and U373-eGFP cells

Direct co-cultures of U87 and U373-eGFP cells were set-up in 6-well plates, into which the cells were seeded at the total density of 10^5^ cells/3ml as five different ratios: 1:0, 1:3, 1:1, 3:1, and 0:1. The growth medium was changed the next day for the growth medium containing TMZ at the final concentration of 750 μM, with growth medium containing 0.5% DMSO as the TMZ control. After 72 h, the cells were trypsinized and the viable (attached) cells ratio determined by FACS analysis (BD FACSCalibur™; BD Biosciences, USA) using the CellQuest software (BD Biosciences, USA).

### Cytokinesis-block micronucleus assay

U87, U251 and U373 cells were seeded into T25 flasks (Corning, NY, USA) at 450 000 cells/flask. The next day, the medium was changed and the cells were exposed to CM of the relevant cell type for 72 h, with cytochalasin B (2 μg/ml) added to the cells after 48 h. After 72 h, the medium was collected separately for later mixing with trypsinized cells. After centrifugation the pelleted cells were incubated in cold hypotonic solution (75 mM KCl) for 5 min, and then fixed with methanol/acetic acid (3:1, v/v). The samples were then put on microscope slides, and stained with acridine orange (20 μg/ml). The micronuclei (MN), nuclear buds (BUD), and nuclear bridges (NPB) in 1000 bi-nucleated cells, were counted per experimental condition, at 400× magnification under a fluorescent microscope (Eclipse 800; Nikon, Japan), according to criteria published by Fenech [51]. Similarly, the numbers of apoptotic/necrotic cells were evaluated per 1000 cells counted.

### 2D – Matrigel invasion assay

The invasion of U87, U251 and U373 cells, either indirectly co-cultured with each other or exposed to the CM (of the relevant cell type), was studied in Matrigel coated Boyden chambers (12-well plates). The upper surfaces of the filter inserts with 12 μm pores, (Costar, Corning Incorporated, NY, USA) were coated with Matrigel (50 μg/insert, Becton Dickinson, Franklin Lakes, USA) in serum-free growth medium. The inserts were dried overnight and reconstituted with 0.2 ml of serum-free growth medium. The cells were seeded in triplicate either into the inserts at a density of 20 000 cells/insert in 0.2 ml of medium, or into the wells in 0.8 ml growth medium or CM. In the controls and when testing the effects of CM, no cells were plated in the bottoms of the wells. After 72 h, the cell inserts were transferred to fresh growth medium, and MTT reagent was added to both chambers. Three hours later, the formazan crystals were collected separately from the upper and lower chamber by centrifugation of the media, and they were dissolved in 200μl of DMSO, pipetted in duplicate into 96-well plates, with the absorbance measured as described above. The invasion was calculated as the percentages of the absorbance in the lower compartment per total absorbance of the both compartments.

### 3D – collagen invasion assay

U87, U251 and U373 spheroids were generated by hanging drops containing 600 cells in 20 μl medium. Upon 3 days the spheroids were washed off and cultured in 10-cm dishes for three more days to reach diameters of 150–300 μm. They were then embedded into 50 μl drops of type I collagen matrix (1.0 mg/ml, BD Bioscience), incubated for 30 min at 37°C, and then covered with 1 ml control growth medium or relevant CM. The spheroid diameters and cell invasion distances were measured under an inverted microscope (Nikon Eclipse Ti; Nikon Germany) using the NIS Elements software (Nikon, Germany) after 1^st^, 3^rd^, and 5^th^ day. The relative invasion was defined as the distance covered by the invading cells from the spheroid, divided by the spheroid radius.

### Gene expression microarrays

Total RNA was isolated from three biological replicates of monocultured and indirectly co-cultured U87 and U373 cells using the Trizol reagent (Invitrogen Limited, Paisley, UK) according to manufacturer instructions. The RNA was purified using RNeasy Mini kit (Qiagen, UK) and the integrity of the RNA confirmed using an Agilent 2100 Bioanalyzer (Agilent Technologies, USA). The RNA samples were labeled with Illumina TotalPrep RNA Amplification kit (Ambion) and hybridized to the HumanWG-6 v3 Expression BeadChip (Illumina). After scanning, image acquisition was performed using the BeadStudio version 3.3.7 software (Illumina). The raw data have been deposited with the NCBI Gene Expression Omnibus (GEO) under the accession code GSE59634.

### Quantitative real-time PCR

cDNA was generated from 1 μg total RNA using High-Capacity cDNA Reverse Transcription kits (Applied Biosystems, USA) in a 50 μl reaction volume, according to the manufacturer protocol. Expression of *Ccl2, Ccl20, Dpp4* and *Egflam* was quantified using real-time quantitative PCR (ABI 7900 HT Sequence Detection System, Applied Biosystems, USA). Real-time PCR reactions were performed using 1:10 dilutions (1 μl/well) of each cDNA, TaqMan Universal PCR Master Mix (Applied Biosystems, USA) and the TaqMan Gene Expression assays (all from Applied Biosystems, USA): *Ccl20* (chemokine (C-C motif) ligand 20), Hs01011368_m1; *Egflam* (EGF-like, fibronecting type III and laminin G domains), Hs00794831_m1; *Dpp4* (dipeptidyl-peptidase 4), Hs00175210_m1, *Bcl2* (B-cell CLL/lymphoma2), Hs00608023_m1; *Bax* (Bcl-2 associated X protein), Hs99999001_m1. Amplification of the GADPH probe (pre-developed Taqman assay no. 4310884E) was performed as the internal control. The SDS v2.2 software was used to analyze the data according to the comparative Ct method (ΔΔCt algorithm).

### Statistical analyses

All of the above experiments were performed in duplicate and were repeated independently at least three times, unless otherwise stated. ANOVA tests (with post-hoc Dunnett's tests) were used to determine the effects of co-cultures and CM on the GBM cells *versus* the controls, and *p* < 0.05 was considered significant. Data are expressed as means ± standard error (SEM).

For the microarrays, the raw data were analyzed using the R Project for Statistical Computing program – version 2.13.2 [53] (http://www.R-project.org), using the lumi [52] and limma [54] packages for data import and quality control, and a robust spline normalization of log2 transformed data. For probe annotation, the Annotate: Annotation for microarrays package, R package version 1.44.0. (http://www.bioconductor.org/packages/release/bioc/html/annotate.html) was used. Data were inspected using hierarchical clustering (HCA) and the empirical Bayes method [55] was used to detect differentially expressed genes. Two lists of differentially expressed genes were generated, one showing the differences between U87 cells co-cultured with U373 cells and U87 cells grown alone (Supplementary Table S1) and the other one showing the differences for co-cultured U373 cells (Supplementary Table S2). Differentially expressed genes (*p*-value < 0.01) of a third comparison between U87 cells and U373 cells with both grown alone (Supplementary Table S3) were functionally analyzed using the Gorilla (Gene Ontology and enRichment anaLysis and visuaLizAtion tool) [16]. BioMine data mining was also performed for the genes of interest [56].

## SUPPLEMENTARY FIGURES AND TABLES








