# Supplementary material for: Heterogeneous glioblastoma cell cross-talk promotes phenotype alterations and enhanced drug resistance
Source: Oncotarget. 2015 Oct 20;6(38):40998–1017. doi: 10.18632/oncotarget.5701 (PMC4747385; doi:10.18632/oncotarget.5701)
Supplement: Supplementary file 1 [file oncotarget-06-40998-s001.pdf]

SUPPLEMENTARY FIGURES AND TABLES

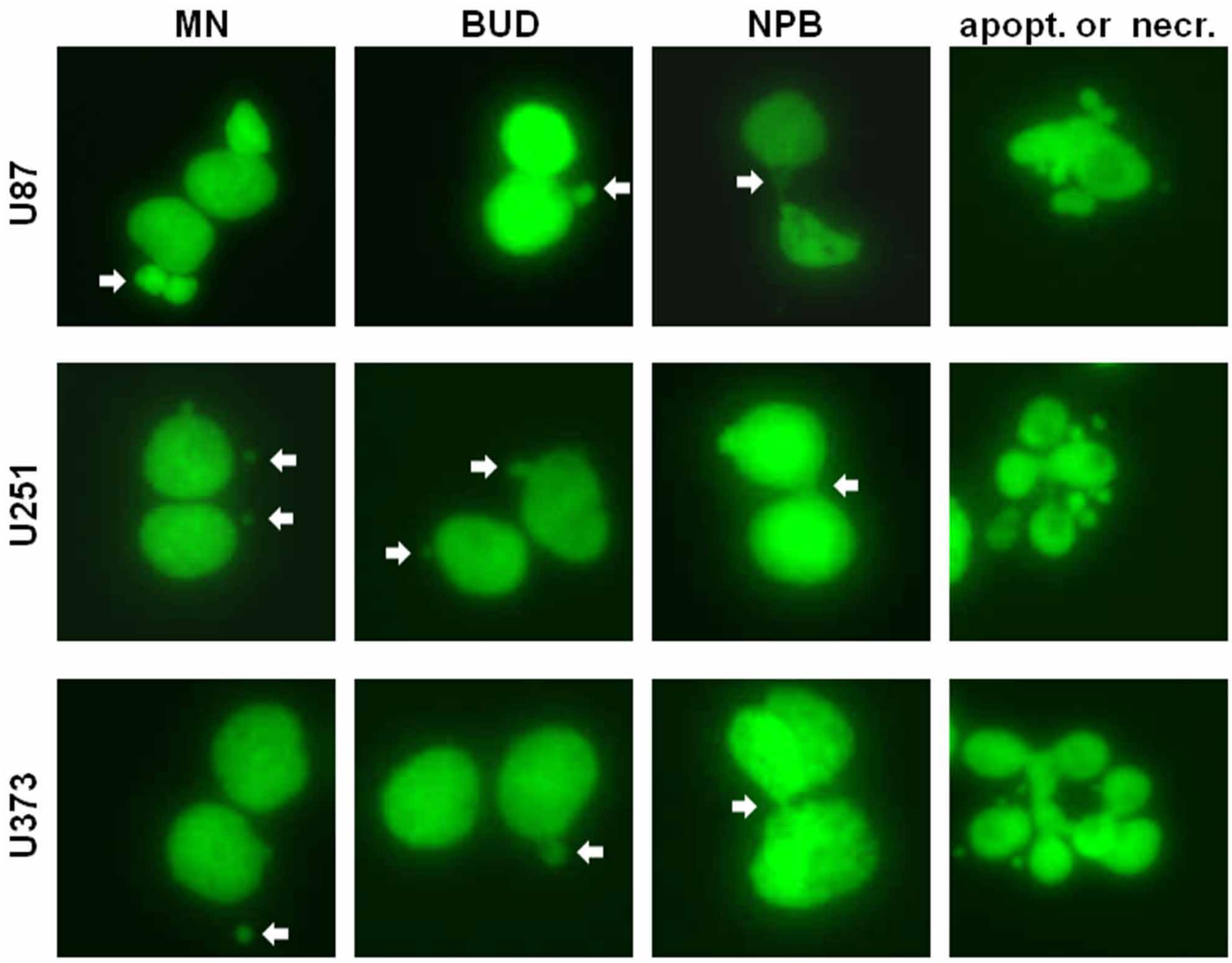

Supplementary Figure S1: Representative images of cell scoring for micronuclei (MN), nucleoplasmic bridges (NPB), and buds (BUD). Cells were scored as binucleated U87, U251 and U373 cells (as indicated) following staining with acridin orange, and indicated by the arrows. The acridin orange staining of the cells also allowed for the apoptotic/necrotic cell detection (4th right column) in all three GBM cell lines (magnification 400 x).

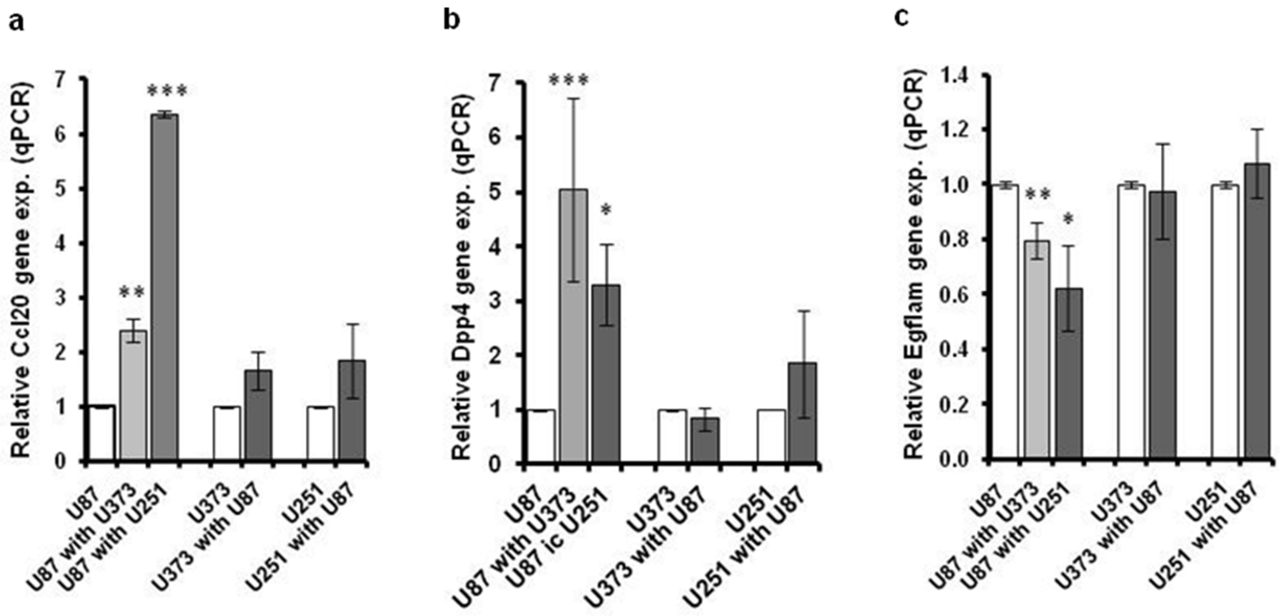

Supplementary Figure S2: qRT-PCR analysis of relative mRNA expression of a. chemokine (C-C motif) ligand 20 (Ccl20), **b.** dipeptidyl-peptidase 4 (Dpp4), **c.** EGF-like, fibronectin type III and laminin G domains (Egflam) in GBM cells grown alone or in indirect co-culture (as indicated). Error bars represent SEM. *p-value < *0.05*.

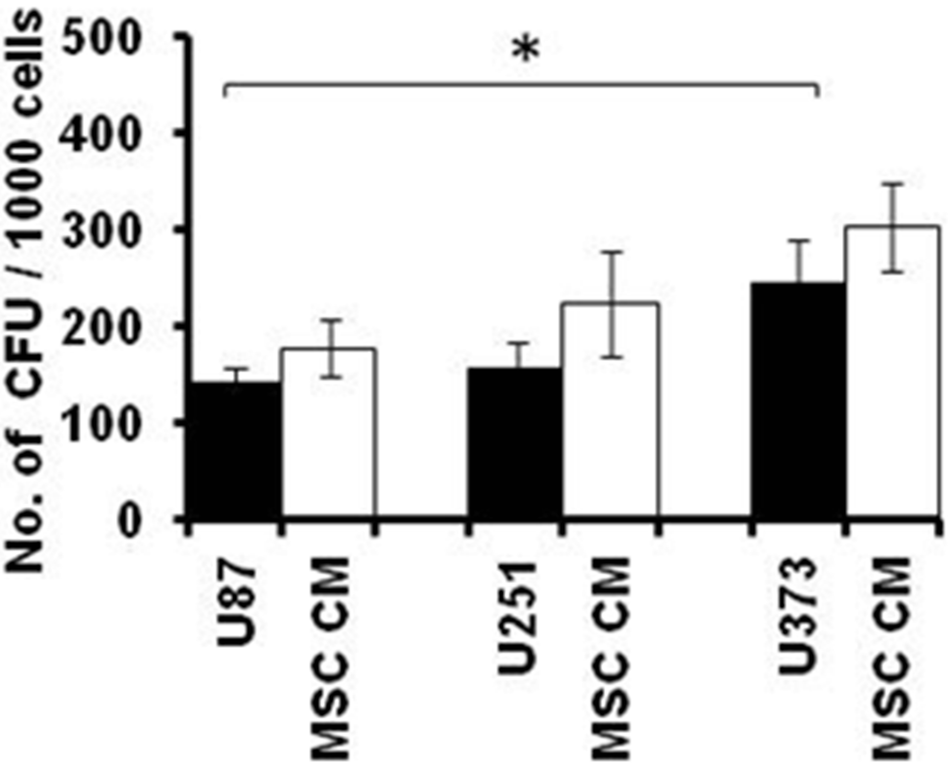

Supplementary Figure S3: Mesenchymal stem cell CM has no effects on CFU of GBM cells. Quantification of CFU formed by U87, U251, and U373 cells grown in normal growth medium and in media conditioned by MSCs (MSC CM). Error bars represent SEM. *p-value < 0.05.

Supplementary Table S1: Differentially expressed genes of U373 cells grown in monoculture when compared to U87 cells grown in monoculture

data of the genes that were regulated (***p***-value <0.01). The up-regulated genes (*positive* log^2FC *values*) are followed by the down-regulated genes (*negative log2FC values*).

Supplementary Table S2: Differentially expressed genes of co-cultured U87 cells

data of all of the genes that were regulated (***p***-value <0.01) in the U87 cells co-cultured with U373 cells, when compared to U87 cell monocultures. The up-regulated genes (*positive* log^2FC *values*) are followed by the down-regulated genes **(***negative* log^2FC *values***)**.

Supplementary Table S3: Differentially expressed genes of co-cultured U373 cells

data of all genes that were regulated (***p***-value < 0.01) in the U373 cells co-cultured with U87 cells, when compared to U373 cell monocultures. The up-regulated genes (*positive* log^2FC *values*) are followed by the down-regulated genes (*negative* log^2FC *values***)**.
